# Radioisotope constraints of Arctic deep water export to the North Atlantic

**DOI:** 10.1038/s41467-021-23877-4

**Published:** 2021-06-16

**Authors:** Lauren E. Kipp, Jerry F. McManus, Markus Kienast

**Affiliations:** 1 Lamont-Doherty Earth Observatory of Columbia University, Palisades, NY USA; 2 Department of Oceanography, Dalhousie University, Halifax, NS Canada; 3 Department of Environmental Science, Rowan University, Glassboro, NJ USA; 4 Department of Earth and Environmental Sciences, Columbia University, New York, NY USA

**Keywords:** Palaeoceanography, Marine chemistry

## Abstract

The export of deep water from the Arctic to the Atlantic contributes to the formation of North Atlantic Deep Water, a crucial component of global ocean circulation. Records of protactinium-231 (^231^Pa) and thorium-230 (^230^Th) in Arctic sediments can provide a measure of this export, but well-constrained sedimentary budgets of these isotopes have been difficult to achieve in the Arctic Ocean. Previous studies revealed a deficit of ^231^Pa in central Arctic sediments, implying that some ^231^Pa is either transported to the margins, where it may be removed in areas of higher particle flux, or exported from the Arctic via deep water advection. Here we investigate this “missing sink” of Arctic ^231^Pa and find moderately increased ^231^Pa deposition along Arctic margins. Nonetheless, we determine that most ^231^Pa missing from the central basin must be lost via advection into the Nordic Seas, requiring deep water advection of 1.1 – 6.4 Sv through Fram Strait.

## Abstract (web summary)

North Atlantic deep water (NADW) formation influences the climate and carbon cycle, but the contribution of Arctic waters is difficult to constrain. Here the authors use Pa/Th proxy measurements to determine the amount of Arctic Ocean water that flows through the Fram Strait and contributes to NADW.

## Introduction

Deep water export from the Arctic to the North Atlantic through the Nordic Seas affects the formation of North Atlantic Deep Water (NADW)^1,2^, a critical component of thermohaline circulation. Because NADW plays a prominent role in the oceanic transport of latent and sensible heat and the sequestration of atmospheric CO_2_, a well-defined record of Arctic contributions to NADW formation is critically important.

The ratio of ^231^Pa (*t*_1/2_ = 3.2 × 10^4^ y) and ^230^Th (*t*_1/2_ = 7.5 × 10^4^ y) in deep-sea sediments can be used as a proxy for deep water circulation and help constrain changes in Arctic outflow. These two naturally occurring radioactive isotopes are produced constantly and ubiquitously in the water column through the decay of uranium isotopes. Due to their long oceanic residence times, uranium isotopes have relatively uniform concentrations and produce ^231^Pa and ^230^Th in a constant ratio of 0.093. Unlike their soluble uranium parents, ^231^Pa and ^230^Th are particle reactive, and their contrasting removal rates by adsorption onto settling particles (scavenging) can be exploited to gain information about ocean circulation and particle fluxes.

Thorium-230 has a high particle affinity, and thus a short oceanic residence time of ~20–40 years^3^. As a result, this isotope is generally removed from solution in the same geographic location as it is produced, such that the inventory of ^230^Th in sediments balances its production in the overlying water column^4^. Protactinium-231 is somewhat more soluble, with an oceanic residence time of ~100–200 years, and can therefore be preferentially affected by lateral transport, including advection, before scavenging and deposition in sediments^3^. This differential scavenging results in sedimentary ^231^Pa/^230^Th ratios that diverge from the water column production ratio of 0.093^3,5–7^. In areas of low particle flux, some ^231^Pa can escape scavenging and be laterally transported, resulting in low ^231^Pa/^230^Th ratios (<0.093) in underlying sediments. In areas of high particle flux, such as ocean margins, a greater proportion of dissolved ^231^Pa and ^230^Th are removed via scavenging (a process called boundary scavenging^5,8^). Because there may be additional ^231^Pa in the water column that was transported from areas of low particle flux, this enhanced removal can result in sedimentary ^231^Pa/^230^Th ratios > 0.093.

**Fig. 1:**
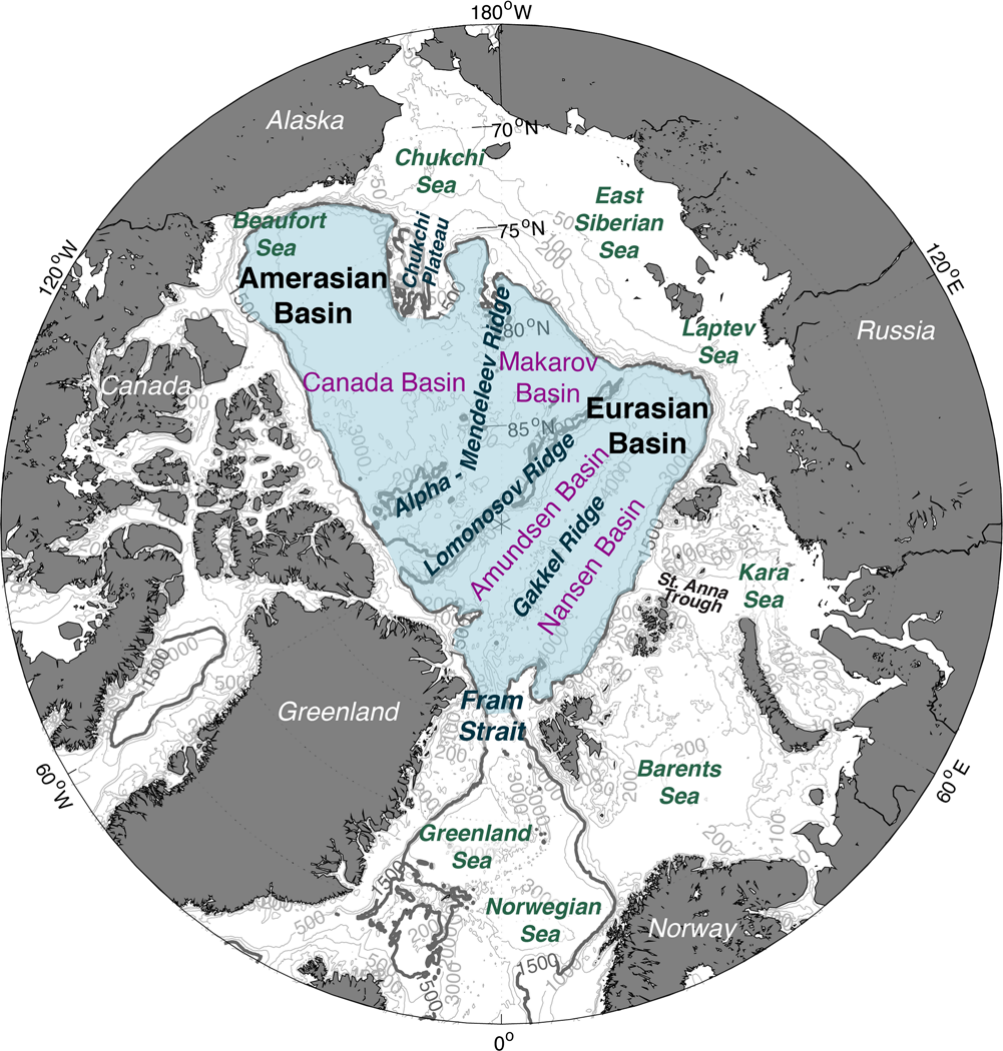
Map of the Arctic Ocean. Blue shading denotes the region considered to be the central basin for the purposes of this study, generally following the 1500 m isobath (bold contour).

Sedimentary ^231^Pa/^230^Th ratios across the central Arctic are low^9–15^, indicating that ^231^Pa is laterally transported away from this region of low particle flux. However, the low spatial resolution of sedimentary observations along the margins of the Arctic basin has made it difficult to determine if the ^231^Pa missing from the central basin is deposited in margin sediments as a result of boundary scavenging or if ^231^Pa is being exported out of the Arctic. The Fram Strait is the only deep conduit in the Arctic, connecting the Eurasian Basin to the North Atlantic through the Nordic Seas (Fig. 1), thus a loss of ^231^Pa from the Arctic indicates southward advection of deep water into the Nordic Seas. Finding the “missing sink” of ^231^Pa is therefore essential to the interpretation of sedimentary ^231^Pa/^230^Th ratios as a proxy for deep water transport out of the Arctic^10,11^.

Here, we expand the geographic coverage of ^231^Pa/^230^Th measurements in Arctic sediments and find slightly elevated ^231^Pa/^230^Th ratios along the margins, consistent with enhanced ^231^Pa deposition via boundary scavenging. Still, a mass balance calculation indicates that most of the ^231^Pa missing from the central basin must be lost via advection into the Nordic Seas. This revised budget is consistent with ^231^Pa loss via the advection of Arctic intermediate and deep waters to the Nordic Seas and North Atlantic, demonstrating the utility of the ^231^Pa/^230^Th ratio as a proxy for past changes in Arctic contributions to NADW. Further, this mass balance provides a geochemical constraint on modern advection through Fram Strait, indicating that the southward flow of Arctic intermediate and deep water is ~1.1–6.4 Sv, averaged over the 100–200 y residence time of this radioisotope.

## Results and discussion

### Coretop ^231^Pa/^230^Th ratios

To improve the Arctic-wide ^231^Pa budget, we have expanded the coverage of ^231^Pa/^230^Th measurements in surface sediments along the margins of the Arctic Ocean (continental slopes on the periphery of the Eurasian and Amerasian Basins) and in the central Canada Basin. Because the majority of our samples were collected from elevated locations near continental margins where sedimentation rates are high, we assume that the ^231^Pa/^230^Th ratios are not appreciably influenced by radioactive decay, bioturbation, or turbidites. Although sedimentation rates are significantly lower in the basins, these assumptions are unlikely to change the final ^231^Pa/^230^Th ratios by more than a few percent (see Supplementary Discussion).

**Fig. 2:**
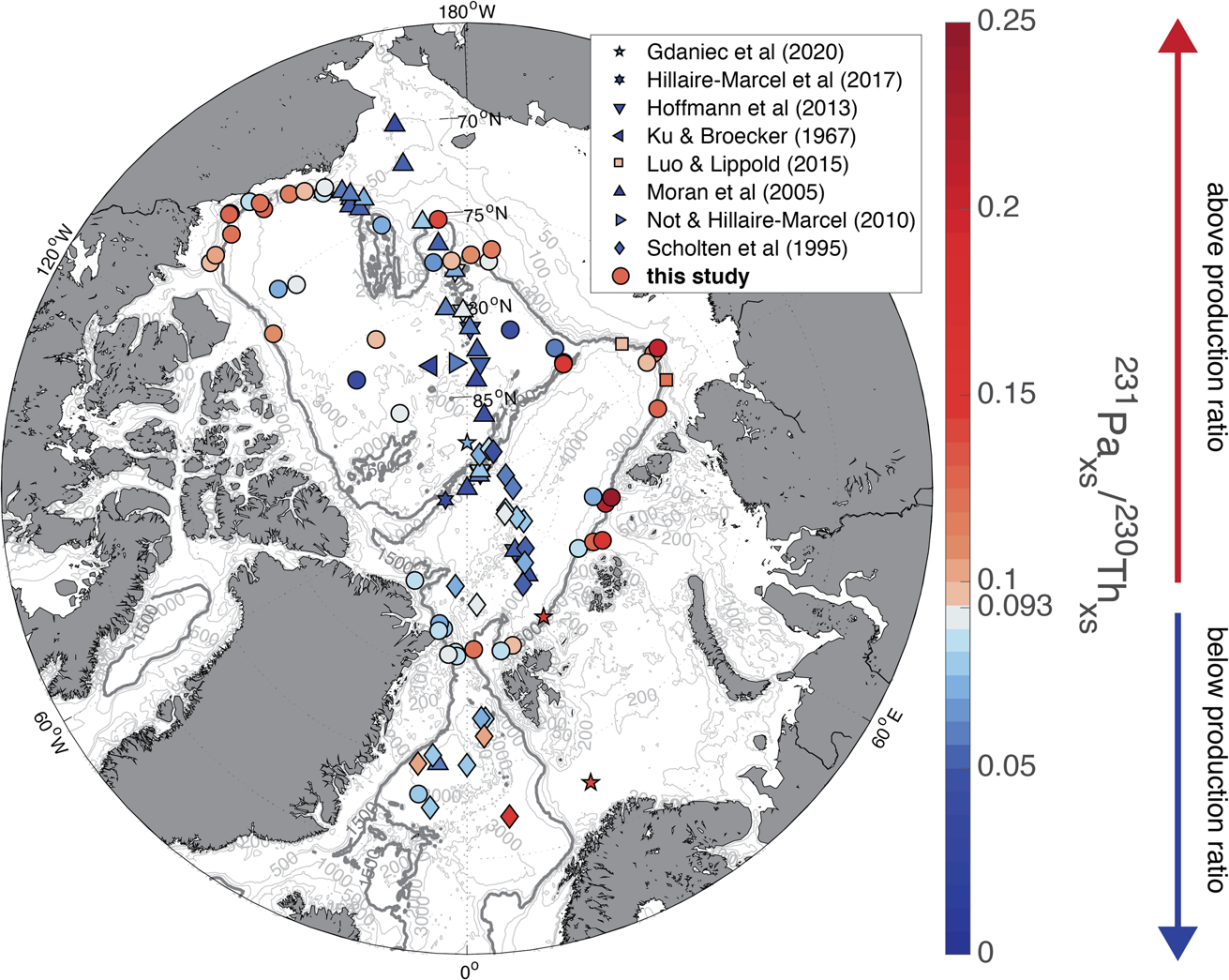
Excess protactinium-231/thorium-230 activity ratios in bulk surface sediments. Symbol shape indicates the data source; new data provided by this study are shown in circles. Orange and red symbols indicate ^231^Pa/^230^Th ratios above the production ratio (0.093), blue symbols indicate ^231^Pa/^230^Th ratios below the production ratio. The 1500 m isobath is shown in bold.

**Fig. 3:**
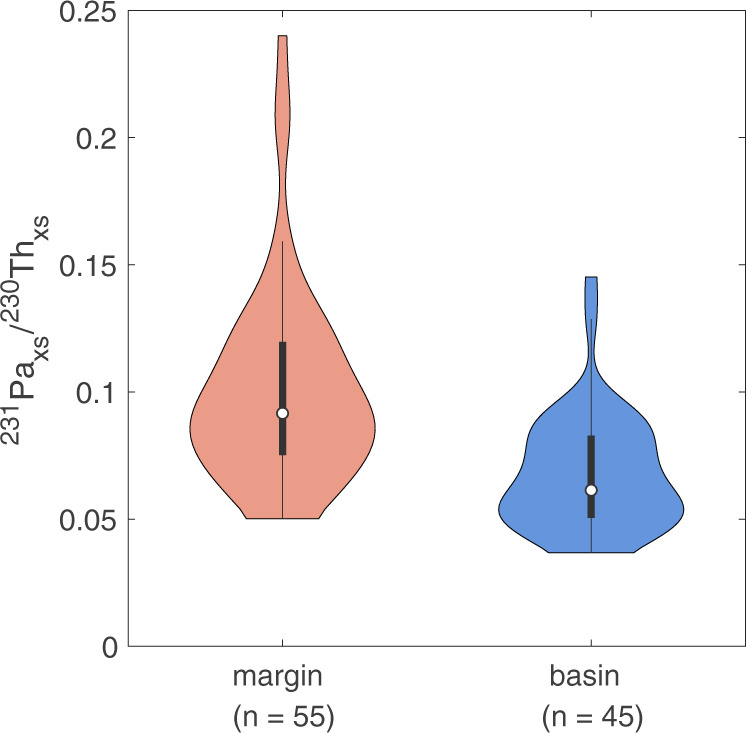
Violin plot of excess ^231^Pa/^230^Th ratios in margin samples (orange) and basin samples (blue). The shape of each plot represents the kernel density estimation, the white dot represents the median, the thick black bar represents the interquartile range, and the thin black line represents the upper and lower adjacent values (±1.5 interquartile range). Wider sections of the plot indicate a higher probability that a data point in the group will have that value, while narrow sections indicate a low probability that the data in the group falls in that range.

^231^Pa/^230^Th ratios in margin sediments are generally higher than those in the basin (Fig. 2). The average ^231^Pa/^230^Th ratio based on previously published data from the central basin^9–15^ was 0.063 ± 0.003 (±SE, *n* = 36); this ratio is not significantly changed by the addition of our new data, increasing slightly to 0.068 ± 0.003 (*n* = 45) (Fig. 3). However, the addition of 40 new samples at the margins notably increases the average margin ratio from 0.077 ± 0.007 (*n* = 15; previous studies^10,15,16^) to 0.101 ± 0.005 (*n* = 55). Because the surface sediment samples are not evenly distributed throughout the study area, we also determined area-normalized averages by gridding the data into equal-area bins (Supplementary Fig. 1). This area-normalization does not change the margin ^231^Pa/^230^Th (0.101 ± 0.005; *n* = 40), and only slightly decreases the basin average to 0.065 ± 0.004 (*n* = 33).

### Protactinium-231 mass balance

The addition of many new margin samples with ^231^Pa/^230^Th ratios near or slightly above the water column production ratio of 0.093 provides evidence of boundary scavenging of ^231^Pa: the enhanced accumulation of ^231^Pa in sediments resulting from higher particle fluxes. To quantify how much of the ^231^Pa missing from the central basin is deposited along the margins, we follow the mass balance approach developed by Luo and Lippold^16^, which first assumes that all of the ^231^Pa missing from the central basin is balanced by boundary scavenging along the margins, in order to determine the expected margin ^231^Pa/^230^Th ratio (^*231*^*Pa/*^*230*^*Th*_*margin*_). This expected ^*231*^*Pa/*^*230*^*Th*_*margin*_ is then compared to the observed ^*231*^*Pa/*^*230*^*Th*_*margin*_ to determine the fraction of ^231^Pa that can be explained by boundary scavenging. Importantly, this method assumes that the inventory of ^230^Th in sediments is balanced by its inventory in the overlying water column (i.e., any changes in the ^231^Pa/^230^Th ratio are due to the addition or removal of ^231^Pa). Previous studies have shown minimal export of ^230^Th from the Arctic, supporting this assumption^10,13,17^.

Comparing the area-normalized average ^231^Pa/^230^Th in the basin (^*231*^*Pa/*^*230*^*Th*_*basin*_; 0.065 ± 0.004) to the expected production ratio of 0.093 and assuming that 100% of the ^230^Th produced is buried in underlying sediments^10,13,17^ indicates that 70 ± 4% of the ^231^Pa produced in the basin is buried there. If all of the missing ^231^Pa is deposited along the margins, the expected ^*231*^*Pa/*^*230*^*Th*_*margin*_ is 0.305 ± 0.024. However, the observed area-normalized average ^*231*^*Pa/*^*230*^*Th*_*margin*_ is 0.101 ± 0.005, thus only 4 ± 1% of the ^231^Pa produced in the basin can be accounted for at the margins, and the remaining 26 ± 2% must be exported to the Nordic Seas.

Recent water column measurements of ^231^Pa support a net export of ^231^Pa through Fram Strait^15^, and previous Arctic-wide assessments of surface sediments (based on very few ^231^Pa/^230^Th measurements along the margins) concluded that ~30–40% of the ^231^Pa produced in the Arctic is exported^10,11^. The greatly improved spatial coverage in this study therefore provides a much better constrained (and slightly decreased) estimate of ^231^Pa leaving the Arctic of ~26%, but upholds the conclusion that advection through Fram Strait is the main sink for the ^231^Pa that is missing from central basin sediments.

### Advection through Fram Strait

While the circulation and fluxes of Arctic surface waters have been the subject of detailed investigation^2,18,19^, less is known about deep water circulation and fluxes. Fram Strait (~2600 m) is the only entry and exit point for intermediate and deep waters; additional water inputs through the Barents Sea (200–300 m) and Bering Strait (~50 m), and outputs through the Canadian Archipelago (150–230 m), are all shallow. Atlantic inflow through Fram Strait is therefore the primary source of intermediate and deep waters, with additional contributions from the subduction of dense water formed through brine formation and winter convection on Arctic shelves^2,20^. Intermediate waters (extending from the halocline down to ~1500–1700 m) circulate cyclonically along the margins of the Eurasian and Amerasian Basins, with pathways following the Lomonosov and Gakkel Ridges in the Eurasian Basin, and recirculating cyclonic loops in the Makarov and Canada Basins^2^. Deep water (>1700 m) circulation pathways are less understood, but are thought to follow a cyclonic circulation from the Eurasian Basin into the Amerasian Basin. Southward flowing cold, dense intermediate and deep waters are transported through Fram Strait and contribute to Denmark Strait Overflow Water and NADW^2,21–23^.

Protactinium-231 is an excellent geochemical tracer of intermediate and deep circulation because ^231^Pa concentrations are low in surface waters (<0.05–0.1 dpm m^−3^ above 500 m)^9,15,24–26^ but increase with depth. A mass balance of ^231^Pa in the Arctic can therefore reasonably exclude surface waters and focus on intermediate and deep water exchange through Fram Strait.

Using surface sediment ratios, we have determined that ~74% of the ^231^Pa produced in the Arctic is buried there, and ~26% must be exported via intermediate and deep water advection. By combining these estimates with water column concentrations, we can determine the amount of intermediate and deep water exchange that must occur through Fram Strait. At steady state, the sources of ^231^Pa to the Arctic Ocean (production of ^231^Pa via radioactive decay and advection of intermediate and deep waters from the Greenland Sea into the Arctic) are balanced by losses (particle scavenging and subsequent deposition in sediments, and advection of intermediate and deep waters out of the Arctic). The advective sources and sinks of ^231^Pa depend on the water fluxes through Fram Strait and the concentrations of ^231^Pa in the Greenland Sea and Arctic Ocean. To our knowledge, there is only one published measurement of ^231^Pa in the Greenland Sea: 0.15 dpm m^−3^ (at 1700 m)^10^. However, similar activities of 0.11–0.16 dpm m^−3^ were measured in Fram Strait Branch water in the Nansen Basin north of the Barents Sea^15^; this is water that has just recently entered the Arctic through Fram Strait. We therefore use a range of 0.11–0.16 dpm m^−3^ for inflowing intermediate and deep waters. Activities in Canada Basin intermediate and deep waters typically increase from ~0.1 dpm m^−3^ around 500 m to ~0.6 dpm m^−3^ near the bottom (>3000 m), while Eurasian Basin intermediate and deep waters increase from ~0.1 to ~0.5 dpm m^−3,^^9,15,24–27^. We therefore assume that average ^231^Pa activities in intermediate and deep waters exported from the Arctic are 0.2–0.3 dpm m^−3^.

Geostrophic estimates of water transport through Fram Strait suggest a net transport of ~2 Sv out of the Arctic^2,28^. However, most of this transport occurs at the surface. Marnela et al.^29^ suggest that the exchange of intermediate and deep waters (defined therein as σ > 28.06) results in a net southward export of ~0.4 Sv. Assuming that the Arctic intermediate and deep water outflow is 0.4 Sv greater than the Greenland Sea inflow, the ^231^Pa mass balance indicates a southward outflow of Arctic waters of 1.1–4.8 Sv, and an inflow of 0.7– 4.4 Sv from the Greenland Sea (minimum and maximum exchanges based on the range in water column ^231^Pa activities). Alternatively, we can assume that the inflow and outflow of intermediate and deep waters are equal; an inverse model of volume fluxes through Arctic gateways suggests that deep water inflow and outflow through Fram Strait are approximately balanced^30^. In this case, the mass balance yields an exchange of 1.3–6.4 Sv. Consequently, an export of 1.1–6.4 Sv of southward flowing Arctic intermediate and deep waters is needed to explain the observed ^231^Pa deficit in Arctic surface sediments.

This estimate is within the lower bounds of the significantly larger range of fluxes inferred previously from geostrophy and ADCP analyses, which range from 1.8 to 18.4 Sv^18,28,29,31^, depending on the density or depth range chosen and the measurement approach. Our geochemistry-based estimate also offers an advantage over more direct volume measurements, because the activities of ^231^Pa reflect conditions integrated over the water column residence time of this isotope (~100–200 years), unlike snapshot measurements. Our ^231^Pa-based estimate could be improved by increasing the number of dissolved ^231^Pa measurements in the Greenland Sea, which would refine the estimate of the northward ^231^Pa flux through Fram Strait. If the ^231^Pa activity of inflowing water is higher than the range estimated here (0.11–0.16 dpm m^−3^), it would increase the flux of southward flowing intermediate and deep waters, and vice-versa.

Export of 1.1– 6.4 Sv from an Arctic Ocean basin volume of 1.1474 × 10^7^ km^3^ (>1500 m, Fig. 1) implies that the residence time of water below 1500 m is 57–331 y. Previously reported residence times for Arctic intermediate waters are on the order of decades^32–39^, while deep water residence time estimates range from ~200 to 600 years in the Amerasian Basin and ~150–300 years in the Eurasian Basin^38–41^. Our estimate includes both deep waters and intermediate waters, thus it is reasonable that our range falls on the lower end of previous estimates for deep water residence times.

The revised Arctic ^231^Pa budget presented herein provides a geochemical constraint on modern circulation in Fram Strait while also providing evidence that the ^231^Pa/^230^Th ratio can be applied as a proxy for past changes in Arctic circulation. Given these improved constraints on the ^231^Pa/^230^Th ratios of Holocene Arctic sediments, future work should focus on developing downcore records to investigate how changing Arctic outflows may have influenced the formation of NADW through time. In particular, further constraints on the amount of Arctic ventilation during the Last Glacial Maximum are needed to resolve conflicting records of persistent deep water export^11,16^ and indications of an isolated, stagnant Arctic basin during this period^42^.

## Methods

### Study region and sample locations

Samples were collected from existing cores archived in multiple international repositories (Woods Hole Oceanographic Institution, USA; U.S. Geological Survey, USA; Institute of Ocean Sciences, Canada; Alfred Wegener Institute, Germany; GEOMAR Helmholtz Centre for Ocean Research Kiel, Germany). Most samples were taken from the top 0 to 1 cm of cores, however in some of the cores collected along the Beaufort shelf and slope (MC-12, MC-21, MC-26, MC-42, MC-46) the top of the core was missing and samples were collected from the shallowest available depth (deepest interval was 4–5 cm).

### Isotopic analyses

Thorium (^230^Th, ^232^Th), protactinium (^231^Pa), and uranium (^234^U, ^238^U) concentrations were measured using isotope dilution and mass spectrometry on an Element 2 inductively coupled plasma mass spectrometer (ICP-MS) at the Lamont-Doherty Earth Observatory of Columbia University. Approximately 100 mg of sediment was spiked with ^229^Th, ^233^Pa, and ^236^U before digestion in HClO_4_, HNO_3_ and HF^43^. Isotopes were separated by co-precipitation with Fe(OH)_3_ followed by anion exchange column chemistry (AG1-X8 anion resin)^44,45^. Reproducibility was assessed using internal standards of homogenized Arctic sediments and North Atlantic sediments; relative standard deviations were <6% for all isotopes. Background contamination was corrected for with Milli-Q water blanks that were digested and analyzed using the same procedure as samples.

Excess ^230^Th and ^231^Pa (^*230*^*Th*_*xs*_, ^*231*^*Pa*_*xs*_) were calculated by subtracting lithogenic ^230^Th and ^231^Pa (^*230*^*Th*_*lith*_, ^*231*^*Pa*_*lith*_) from total ^230^Th and ^231^Pa (^*230*^*Th*_*tot*_, ^*231*^*Pa*_*tot*_), respectively (all in activity per sample mass, or dpm/g):

1 $${}^{230}T{h}_{xs}={}^{230}T{h}_{tot}-{}^{230}T{h}_{lith}$$

2 $${}^{231}P{a}_{xs}={}^{231}P{a}_{tot}-{}^{231}P{a}_{lith}$$

This corrects for ^230^Th and ^231^Pa sourced from continental material rather than produced via U decay in the water column. The lithogenic ^230^Th activity was determined for each sample using the activity ratio of ^238^U and ^232^Th in lithogenic material, *(*^*238*^*U/*^*232*^*Th)*_*lith*_, and the measured activity of ^232^Th (dpm/g), which is solely of lithogenic origin^46^:

3 $${\,\!}^{230}Th_{lith}=({\,\!}^{238}U/{\,\!}^{232}Th)_{lith}{\ast} {\,\!}^{232}Th$$

Similarly, lithogenic ^231^Pa activity was calculated using Eq. 3 multiplied by 0.046, the natural ^235^U/^238^U activity ratio:

4 $${\,\!}^{231}P{a}_{lith}=0.046\ast ({\,\!}^{238}U/{\,\!}^{232}Th)_{lith}\ast {\,\!}^{232}Th$$

This approach assumes that ^238^U and ^235^U are in secular equilibrium with ^230^Th and ^231^Pa, respectively, and that *(*^*238*^*U/*^*232*^*Th)*_*lith*_ is known. Previous pan-Arctic studies have used *(*^*238*^*U/*^*232*^*Th)*_*lith*_ ratios between 0.5 and 0.7, most commonly 0.6 ± 0.1^10,11,15,16^. However, the Amerasian Basin is likely to have a higher average *(*^*238*^*U/*^*232*^*Th)*_*lith*_ than the Eurasian Basin due to the presence of detrital carbonates in the Canadian Shield^47^, which can have ^238^U/^232^Th ratios as high as 4^48^. The bulk sediment ^238^U/^232^Th ratios measured in our samples were higher in the Canada Basin compared to the Makarov, Nansen, and Amundsen Basins, in line with previous studies^10,14^ (Supplementary Fig. 2). Due to this geographic variation, we use a ^238^U/^232^Th ratio of 0.7 ± 0.1 to correct for lithogenic inputs in the Canada Basin, and a ratio of 0.6 ± 0.1 to correct for lithogenic inputs elsewhere. Using a higher lithogenic ratio causes the ^231^Pa_xs_/^230^Th_xs_ ratio (referred to hereafter and in the main text as ^231^Pa/^230^Th ratio) to increase slightly.

Authigenic ^230^Th and ^231^Pa (produced through the in-situ decay of U that has precipitated in reducing sediments) were not accounted for because we assume a surface sediment age of 0 ka, thus no time has elapsed during which ^230^Th and ^231^Pa could have accumulated from authigenic U decay (see Supplementary Discussion).

Errors on individual ^231^Pa/^230^Th ratios are reported as 2$\sigma$. As the errors on the ICP measurements are only a few percent, the largest sources of error are the sizeable lithogenic correction and the error on the detrital *(*^*238*^*U/*^*232*^*Th)*_*lith*_ ratio. Errors on averaged ^231^Pa/^230^Th ratios are reported as standard error (SE = ${\rm{\sigma }}/\sqrt{{\rm{n}}}$), to reflect increased confidence in the value as data coverage improves.

To compare our results with published data, we recalculated previously reported ^231^Pa/^230^Th ratios in the Canada Basin using a *(*^*238*^*U/*^*232*^*Th)*_*lith*_ ratio of 0.7 when possible^10,11,15^. For two samples in the Canada Basin near the Alpha-Mendeleev Ridge, total ^231^Pa and ^230^Th were not reported and excess activities were based on measured uranium activities rather than the *(*^*238*^*U/*^*232*^*Th)*_*lith*_ ratio^12,14^.

### Margin-basin designation

Samples were separated into central basin and margin bins by eye based on proximity to the continental slope. As ridges are the second largest physiographic province in the Arctic (preceded by continental shelves)^49^, it is impossible to use a cutoff based on depth or distance to an isobath; this approach would incorrectly place many of the samples collected from ridges into the margin category. Sensitivity tests were performed to ensure that samples located in areas that could be considered part of either bin (e.g., near the Chukchi Plateau, Lomonosov Ridge near Siberia) did not change the conclusions of this study. In all cases, moving these samples from one bin to another changed the average ^231^Pa/^230^Th ratios of the margin and basin bins by <2%. Samples located in the Greenland Sea and on the shallow shelf (<400 m) were not included in either the basin or margin groups (*n* = 4).

### Equal-area grid

Area-normalized averages were determined by gridding the data into equal-area bins (Supplementary Fig. 1). Each bin has a height of 1-degree, and an area of ~12,400 km^2^. The longitude range of each bin grows larger toward the pole, as the number of bins per degree of latitude decreases. The assignment of each bin as basin or margin is based on the location of the samples inside the bin, rather than the center of the bin. Changing the bin size does not notably affect the results; doubling the height of each bin from 1 to 2-degrees of latitude increases the average margin ^231^Pa/^230^Th ratio by 5% and does not change the average basin ratio.

### Sediment mass balance of protactinium-231

If all the ^231^Pa lost from the central basin was balanced by boundary scavenging along the margins (i.e., if the Arctic had no deep water connection to the Atlantic), then the deficit of ^231^Pa reflected in ^231^Pa/^230^Th ratios measured in the central basin (^*231*^*Pa/*^*230*^*Th*_*basin*_) multiplied by the volume of the central basin (*V*_*basin*_) must be balanced by ^231^Pa excesses in the margin ratios (^*231*^*Pa/*^*230*^*Th*_*margin*_) multiplied by the volume of the margin box (*V*_*margin*_)^16^:

5 $$({\,\!}^{231}Pa/{\,\!}^{230}T{h}_{margin}-0.093)\ast {V}_{margin}=(0.093-{}^{231}Pa/{\,\!}^{230}T{h}_{basin})\ast {V}_{basin}$$

In reality, not all of the ^231^Pa missing from the central basin is deposited along the margins, but some is exported to the Nordic Seas. To determine the fraction of missing ^231^Pa that can be found at the margins (*f*), the observed margin ratio is compared to the expected value determined in Eq. 5^:16^

6 $$f=\frac{({{\rm{measured}}}^{231}{Pa}{{/}^{230}{Th}}_{{margin}}-0.093)}{({{\rm{expected}}}^{231}{Pa}{{/}^{230}{Th}}_{{margin}}-0.093)}\times 100 \%$$

The volume of the central basin was determined by drawing a polygon following the 1500 m isobath but including the ridges, such that regions shallower than 1500 m inside the polygon were considered part of the basin (Fig. 1). The volume inside the polygon (*V*_*basin*_) is 1.1474 × 10^7^ km^3^ (based on the ETOPO2_v2 2 min gridded bathymetry from NOAA; www.ngdc.noaa.gov/mgg/global/etopo2.html). The volume of the margins (*V*_*margin*_; shelves and slope down to 1500 m) was determined to be 0.1516 × 10^7^ km^3^ by subtracting the basin volume from the total Arctic volume (1.2990 × 10^7^ km^3^)^50^.

### Water column mass balance of protactinium-231

The mass balance of ^231^Pa in the Arctic water column is summarized in Eq. 7:

7 $${V}_{AO}\ast \partial {\,\!}^{231}Pa/\partial t={\beta }_{231}{V}_{AO}-{S}_{231}{V}_{AO}+({\,\!}^{231}P{a}_{GS}){F}_{GS}-({\,\!}^{231}P{a}_{AO}){F}_{AO}$$

Where *β*_*231*_ is the production rate of ^231^Pa (dpm m^−3^ y^−1^), S_231_ is the scavenging rate of ^231^Pa (dpm m^−3^ y^−1^), ^*231*^*Pa*_*GS*_ and ^*231*^*Pa*_*AO*_ are the activities of ^231^Pa in the Greenland Sea and Arctic Ocean, respectively (dpm m^−3^), *F*_*GS*_ and *F*_*AO*_ are the northward and southward water fluxes through Fram Strait, respectively (m^3^ y^−1^), *V*_*AO*_ is the total volume of the Arctic Ocean^50^, and ∂^*231*^*Pa*/ ∂*t* is the change in Arctic ^231^Pa concentration through time. At steady state, ∂^*231*^*Pa*/ ∂*t* is zero. Based on our ^231^Pa sediment mass balance, *S*_*231*_ is equal to 0.74 *β*_*231*_. Because both *F*_*GS*_ and *F*_*AO*_ are unknowns, another equation is needed to solve for the volume exchange through Fram Strait. If we assume that the inflow and outflow of intermediate and deep waters is the same (*F* = *F*_*GS*_ = *F*_*AO*_), Eq. 7 then simplifies to:

8 $$F=0.26{\beta }_{231}{V}_{AO}/({\,\!}^{231}P{a}_{AO}-{\,\!}^{231}P{a}_{GS})$$

If we assume a net southward export of 0.4 Sv (1.26 × 10^13^ m^3^ y^−1^)^29^, then *F*_*AO*_ = *F*_*GS*_ + 1.26 × 10^13^ m^3^ y^−1^ and Eq. 9 can be solved for *F*_*AO*_:

9 $${F}_{AO}=({\,\!}^{231}P{a}_{GS}\ast 1.26\times {10}^{13}-0.26{\beta }_{231})/({\,\!}^{231}P{a}_{GS}-{\,\!}^{231}P{a}_{AO})$$

## Supplementary information

Supplementary Information

Supplementary Data 1

Description of additional supplementary files

## Data Availability

The data that support the findings of this study are available through the World Data Service for Paleoclimatology (https://www.ncdc.noaa.gov/paleo-search/study/31932) and in Supplementary Dataset 1.
