# Supplementary material for: Radioisotope constraints of Arctic deep water export to the North Atlantic
Source: Nat Commun. 2021 Jun 16;12:3658. doi: 10.1038/s41467-021-23877-4 (PMC8209033; doi:10.1038/s41467-021-23877-4)
Supplement: Supplementary file 1 — Supplementary Information [file 41467_2021_23877_MOESM1_ESM.pdf]

Supplementary Information for:

Radioisotope constraints of Arctic deep water export to the North Atlantic

Lauren E. Kipp[1,2,3*], Jerry F. McManus[1,4], Markus Kienast[2]

[1] Lamont-Doherty Earth Observatory of Columbia University, Palisades, New York, USA
[2] Department of Oceanography, Dalhousie University, Halifax, Nova Scotia, Canada
[3] Department of Environmental Science, Rowan University, Glassboro, New Jersey, USA
[4] Department of Earth and Environmental Sciences, Columbia University, New York, NY, USA

[*]Corresponding author: kipp@rowan.edu

Supplementary Discussion

Surface sediment age assumption

Because most of our samples were collected on margins where sedimentation rates are high (e.g. 200 – >1,000 cm/ka on the Chukchi and Beaufort margins [1,2]), the assumption that surface sediments have an age of 0 ka is unlikely to bias the calculation of $^{231}Pa/^{230}Th$ ratios. In other areas along the Amerasian Basin margin, sedimentation rates are typically ~2 – 6 cm/ka [3,4], which would correspond to a maximum age of 0.5 ka for the top 1 cm and change the resulting $^{231}Pa/^{230}Th$ ratio by <1%. For this comparison, authigenic ^{230}Th was calculated using Equation 1, where A_{230Th}^{auth} is authigenic ^{230}Th, A_{238U}^{auth} is authigenic ^{238}U, $\left(\frac{A_{234U}}{A_{238U}}\right)_{SW}$ is the $^{234}U/^{238}U$ activity ratio in seawater (1.1468), λ_{230} is the ^{230}Th decay constant, and λ_{234} is the ^{234}U decay constant[5].

$$A_{230Th}^{auth} = A_{238U}^{auth} * \left[\left(1 - e^{-\lambda_{230}*t}\right) + \frac{\lambda_{230}}{\lambda_{230}-\lambda_{234}} * \left(e^{-\lambda_{234}*t} - e^{-\lambda_{230}*t}\right) * \left(\left(\frac{A_{234U}}{A_{238U}}\right)_{SW} - 1\right)\right] \qquad (1)$$

In the ice-covered central basin, sedimentation rates as low as 0.1 – 0.2 cm/ka have been reported [1,6–8], corresponding to a mean age of up to 5 ka for the top 1 cm and a decrease of 5.8% in the final $^{231}Pa/^{230}Th$ ratio. However, these central Arctic sedimentation rates may be biased low by inadequate age models; Backman et al. [9] present evidence that sedimentation rates are likely on the scale of cm/ka across the Arctic.

Bioturbation within the surface mixed layer could also impact $^{231}Pa/^{230}Th$ ratios by diluting younger surface sediments with older sediments containing less ^{231}Pa and ^{230}Th as a result of radioactive decay. Because ^{231}Pa has a shorter half-life than ^{230}Th, older sediments will have a lower $^{231}Pa/^{230}Th$ ratio. This is likely to have a negligible impact along the margins due to high sedimentation rates (i.e. ^{230}Th and ^{231}Pa in the top few cm of sediments will not have appreciably decayed, thus the $^{231}Pa/^{230}Th$ ratio will not be impacted). However, bioturbation in the basin, where there are very low sedimentation rates, could bias the average $^{231}Pa/^{230}Th$ toward lower values and cause an over-estimation of the amount of ^{231}Pa leaving the Arctic. Moran et al.[11] investigated bioturbation in central Arctic sediments and concluded that this process does not impact $^{231}Pa/^{230}Th$ ratios on a basin-wide scale. Further, mixed layer depths in Arctic sediments are shallow (1 – 3 cm)[12], so bioturbation is unlikely to change the final $^{231}Pa/^{230}Th$ ratio by a large percentage (see above).

Turbidites contribute to sediment accumulation in the Arctic, particularly in the Canada Basin[13,14]. Turbidites could impact $^{231}Pa/^{230}Th$ ratios by enhancing scavenging of both isotopes in the basin, thereby increasing $^{231}Pa/^{230}Th$ ratios by allowing less ^{231}Pa to be lost via advection to the margins. This would serve to decrease the difference between basin and margin $^{231}Pa/^{230}Th$ ratios and cause an under-estimation of the amount of ^{231}Pa impacted by boundary scavenging, though it would not impact the calculation of how much ^{231}Pa is leaving the Arctic. Because we see a clear distinction between margin and basin in our sample set, we conclude that turbidites are not significantly biasing our average ratios. The large geographic distribution of basin samples and the fact that many of the samples were collected from mid-ocean ridges also reduce the probability that turbidites have impacted our results.

Bulk $^{238}U/^{232}Th$ ratios

The bulk sediment $^{238}U/^{232}Th$ ratios measured in our samples ranged from 0.54 to 1.17, with higher values in the Canada Basin (average of 0.88, n = 27) compared to the Makarov (0.69, n = 4), Nansen (0.72, n = 20), and Amundsen (0.54, n = 1) Basins (Supplementary Figure 2). Similar ratios have previously been observed along the Canada Basin margin: Moran et al.[11] reported values between 0.64 and 1.10 (average of 0.85) on the Chukchi shelf and slope, and Not and Hillaire-Marcel[6] observed ratios between 0.42 and 1.03 on the Mendeleev Ridge.

Coretop $^{231}Pa/^{230}Th$ ratios

The $^{231}Pa/^{230}Th$ ratios measured along the Beaufort margin are 0.070 – 0.128, higher than those previously measured by Moran et al.[11] on the neighboring Chukchi Sea slope. The increase in $^{231}Pa/^{230}Th$ from west to east around the Canada Basin may be due in part to the outflow of the Mackenzie River, which carries the highest suspended sediment load of any Arctic river[15,16] and discharges into the Beaufort Sea around 135ºW. Sedimentation rates in cores near the mouth of the Mackenzie exceed 1,000 cm/ka [2], and these high particle fluxes will increase the removal of ^{231}Pa from the water column. Nonetheless, high sedimentation rates (~100-300 cm/ka) have also been reported on the Chukchi Slope[1,17], so an enhanced removal of ^{231}Pa would be expected in this region as well.

Particle composition is another possible explanation for the difference between the two regions. As ^{231}Pa is preferentially scavenged by opal[18,19], higher biogenic silica concentrations in the Beaufort Sea could contribute to higher $^{231}Pa/^{230}Th$ ratios. Generally, biogenic silica is a minor component of central Arctic sediments[20] and water column particles[21], but the flux of silica from the Mackenzie River may enhance diatom production in the Beaufort Sea[22], increasing the flux of biogenic silica to sediments[23].

The highest $^{231}Pa/^{230}Th$ ratios measured in this study were found on the Kara Sea slope near the St. Anna Trough. After transiting the Barents and Kara Seas, Atlantic-derived water subducts through this trough to become Arctic Intermediate Water[24]. The high $^{231}Pa/^{230}Th$ ratios may result from the deposition of diatom frustules (and thus biogenic silica) in this area[25] and/or scavenging by benthic nepheloid layers as water moves through the trough[26]. The individual samples in this region have large errors (e.g. 0.240±0.291 and 0.209±0.109, $\pm 2\sigma$), but the similarity of $^{231}Pa/^{230}Th$ ratios in nearby samples lends confidence to the overall trend. Ratios on the nearby Laptev Sea slope were also above the production ratio (0.098 – 0.206), in good agreement with neighboring samples measured by Luo and Lippold[27] (0.098 – 0.120).

The lowest margin ratios were found north of Greenland; this area is ice-covered for most of the year, so low particle fluxes likely result in low boundary scavenging in this area. Nonetheless, the ratios are slightly higher than those in the central basin, indicating slightly increased scavenging near the margin.

In the Canada Basin, $^{231}Pa/^{230}Th$ ratios were generally below the production ratio, with the exception of one sample with a ratio of 0.095±0.002. Ratios were low in the Makarov Basin (~0.05), but increased on the Lomonosov Ridge (~0.14). The low ratios across the Amerasian Basin are in agreement with water column results from recent Arctic GEOTRACES cruises (GN01, GN04), which show that suspended particles also have $^{231}Pa/^{230}Th$ ratios below 0.093[21,28].

The higher ratios on the Lomonosov Ridge may reflect increased ^{231}Pa scavenging driven by proximity to the productive margin; sediment traps indicate that opal is a significant portion (~20-40%) of the mass flux in this area in the summer[29]. Dissolution contributes to a downward decrease in opal flux[29], thus the low $^{231}Pa/^{230}Th$ ratio in the neighboring Makarov Basin sample (collected at 2472 m) likely reflects this change in composition.

Regardless of the mechanisms driving slight differences between neighboring samples, the overall observation of higher $^{231}Pa/^{230}Th$ ratios along the margins is robust. A students t-test was used to determine that the margin and basin groupings of samples were statistically different at the >99.99% confidence level.

Mass balance sensitivity analysis

Using the non-area normalized average $^{231}Pa/^{230}Th_{basin}$ value (0.068±0.003) instead of the area-normalized average $^{231}Pa/^{230}Th_{basin}$ (0.065±0.004) does not significantly change the mass balance. In this case, 73±4% of ^{231}Pa produced in the central basin is buried there, and the expected $^{231}Pa/^{230}Th_{margin}$ is 0.285±0.020. Comparing this to the non-area normalized average $^{231}Pa/^{230}Th_{margin}$ (0.101±0.005) indicates that 4±1% of the remaining ^{231}Pa is found at the margins, while 23±1% leaves the Arctic. Similarly, this calculation is not very sensitive to the isobath chosen for the volume determination: using the 2500 m isobath as the basis for the basin volume polygon instead of 1500 m results in 7±1% of the missing ^{231}Pa buried at the margins and 23±2% leaving the Arctic (the amount buried in the central basin is unchanged because $^{231}Pa/^{230}Th_{basin}$ and $^{231}Pa/^{230}Th_{margin}$ are not dependent on the chosen isobath). Our conclusion that 4-7% of the missing ^{231}Pa is found along the Arctic margins is similar to the estimate of Luo and Lippold[27], who found that 10% of the ^{231}Pa missing from the Eurasian Basin could be accounted for along the Eurasian margins.

As an alternative way to calculate how much ^{231}Pa is leaving the Arctic, we compared the $^{231}Pa/^{230}Th$ ratio of the Arctic as a whole to the production ratio of 0.093. A pan-Arctic weighted average ($^{231}Pa/^{230}Th_{Arctic}$) was determined by multiplying the basin and margin averages by the percent of total Arctic volume (V_{Arctic}) found in each box:

$$^{231}Pa/^{230}Th_{Arctic} = (^{231}Pa/^{230}Th_{margin} * (V_{margin}/V_{Arctic})) + (^{231}Pa/^{230}Th_{basin} * (V_{basin}/V_{Arctic})) \qquad (2)$$

If the area-normalized averages are used, $^{231}Pa/^{230}Th_{Arctic}$ is 0.069±0.004, indicating that 26±4% of the ^{231}Pa produced in the Arctic is exported to the Nordic Seas. If non-area normalized averages are used, $^{231}Pa/^{230}Th_{Arctic}$ is 0.072±0.003, and 23±3% of the ^{231}Pa escapes the Arctic.

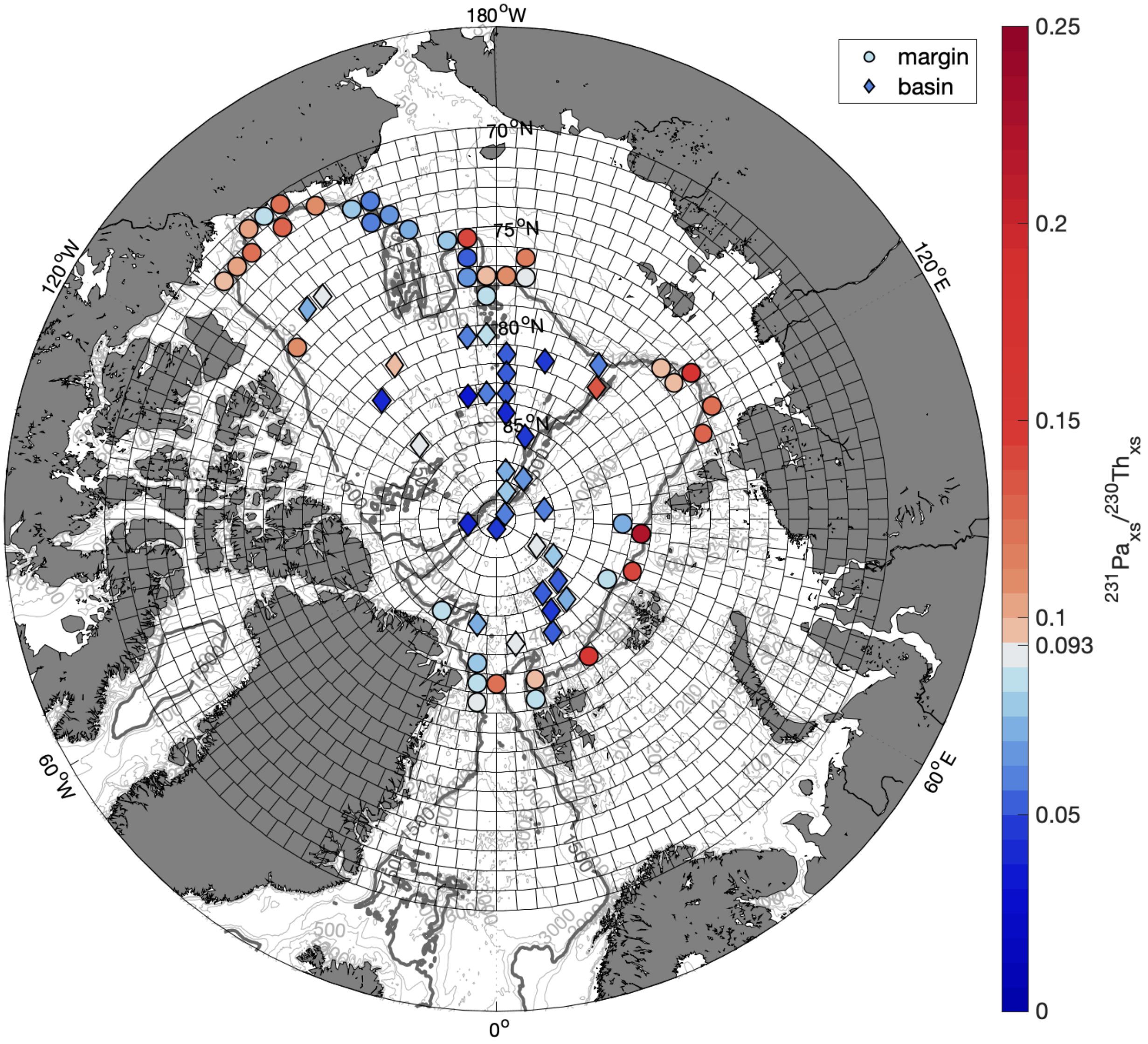

Supplementary Figure 1. Average excess $^{231}Pa/^{230}Th$ ratios in each bin of an equal-area grid. The categorization of each bin as margin (circles) or basin (diamonds) was based on the actual location of the data points in each bin (see Figure 2) rather than the center of the bin. Orange and red symbols indicate $^{231}Pa/^{230}Th$ ratios above the production ratio (0.093), blue symbols indicate $^{231}Pa/^{230}Th$ ratios below the production ratio. The 1500 m isobath is shown in bold. The data used to determine the average ratios derive from this study as well as previously published results; see Figure 2 legend for a list of the data sources.

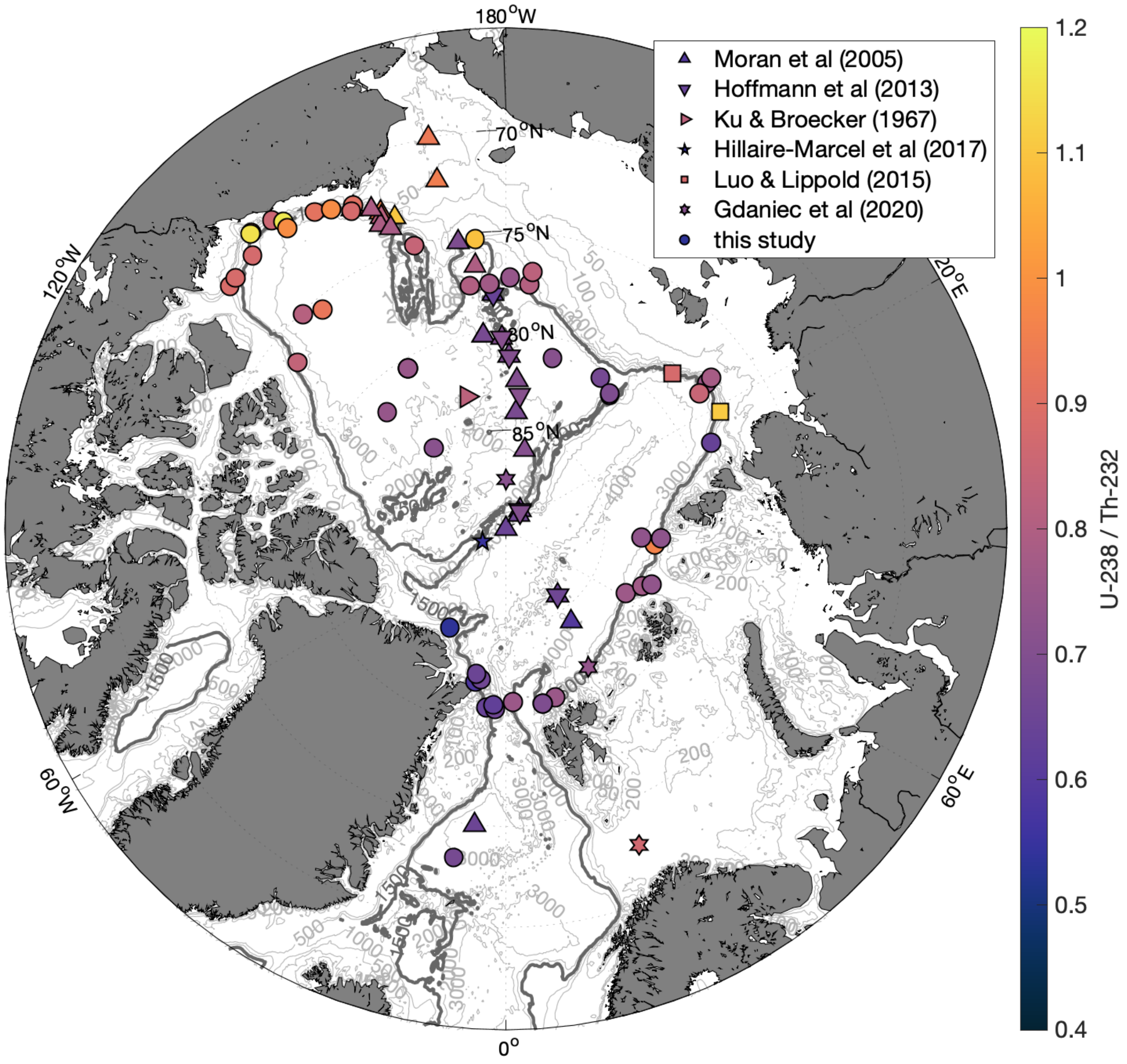

Supplementary Figure 2. Bulk uranium-238/thorium-232 activity ratios in surface sediments. Symbol shape indicates the data source; new data provided by this study are shown in circles. The 1500 m isobath is shown in bold.

Supplementary References

1. Huh, C. A., Pisias, N. G., Kelley, J. M., Maiti, T. C. & Grantz, A. Natural radionuclides and plutonium in sediments from the western Arctic Ocean: Sedimentation rates and pathways of radionuclides. *Deep. Res. Part II Top. Stud. Oceanogr.* **44**, 1725–1743 (1997).
2. Keigwin, L. D. *et al.* Deglacial floods in the Beaufort Sea preceded Younger Dryas cooling. *Nat. Geosci.* **11**, 599–604 (2018).
3. Polyak, L. & Jakobsson, M. Quaternary Sedimentation in the Arctic Ocean: Recent Advances and Further Challenges. *Oceanography* **24**, 52–64 (2011).
4. Stein, R., Matthiessen, J. & Niessen, F. Re-Coring at Ice Island T3 Site of Key Core FL-224 (Nautilus Basin, Amerasian Arctic): Sediment Characteristics and Stratigraphie Framework. *Polarforschung* **79**, 81–96 (2009).
5. Costa, K. M. *et al.* ^{230}Th Normalization: New Insights on an Essential Tool for Quantifying Sedimentary Fluxes in the Modern and Quaternary Ocean. *Paleoceanogr. Paleoclimatology* **35**, (2020).
6. Not, C. & Hillaire-Marcel, C. Time constraints from ^{230}Th and ^{231}Pa data in late Quaternary, low sedimentation rate sequences from the Arctic Ocean: An example from the northern Mendeleev Ridge. *Quat. Sci. Rev.* **29**, 3665–3675 (2010).
7. Hillaire-Marcel, C. *et al.* A New Chronology of Late Quaternary Sequences From the Central Arctic Ocean Based on "Extinction Ages" of Their Excesses in ^{231}Pa and ^{230}Th. *Geochemistry, Geophys. Geosystems* **18**, 4573–4585 (2017).
8. Ku, T.-L. & Broecker, W. S. Rates of sedimentation in the Arctic ocean. *Prog. Oceanogr.* **4**, 95–104 (1967).
9. Backman, J., Jakobsson, M., Løvlie, R., Polyak, L. & Febo, L. A. Is the central Arctic Ocean a sediment starved basin? *Quat. Sci. Rev.* **23**, 1435–1454 (2004).
10. de Vernal, A. *et al.* Natural variability of the Arctic Ocean sea ice during the present interglacial. *Proc. Natl. Acad. Sci.* **117**, 26069–26075 (2020).
11. Moran, S. B. *et al.* ^{231}Pa and ^{230}Th in surface sediments of the Arctic Ocean: Implications for ^{231}Pa/^{230}Th fractionation, boundary scavenging, and advective export. *Earth Planet. Sci. Lett.* **234**, 235–248 (2005).
12. Clough, L. M. *et al.* Bioturbation, biomass, and infaunal abundance in the sediments of the Arctic Ocean. *Deep Sea Res. Part II.* **44**, 1683-1704. (1997).
13. Grantz, A. *et al.* Character, paleoenvironment, rate of accumulation, and evidence for seismic triggering of Holocene turbidites, Canada Abyssal Plain, Arctic Ocean. *Mar. Geol.* **133**, 51–73 (1996).
14. Darby, D. A., Naidu, A. S., Mowatt, T. C. & Jones, G. A. Sediment composition and sedimentary processes in the Arctic Ocean. in *The Arctic seas* (ed. Herman, Y.) 657–720 (Springer, 1989).
15. Carson, M. A., Jasper, J. N. & Conly, F. M. Magnitude and Sources of Sediment Input to the Mackenzie Delta, Northwest Territories, 1974 – 94. *Arctic* **51**, 116–124 (1998).
16. Holmes, R. M. *et al.* A circumpolar perspective on fluvial sediment flux to the Arctic ocean. *Global Biogeochem. Cycles* **16**, (2002).
17. Darby, D. A. *et al.* The role of currents and sea ice in both slowly deposited central Arctic and rapidly deposited Chukchi-Alaskan margin sediments. *Glob. Planet. Change* **68**, 58–72 (2009).

18. Walter, H.-J., Rutgers van der Loeff, M. M. & François, R. Reliability of the ^{231}Pa /^{230}Th Activity Ratio as a Tracer for Bioproductivity of the Ocean. in *Use of Proxies in Paleoceanography* 393–408 (Springer Berlin Heidelberg, 1999).
19. Moran, S. B. *et al.* Dissolved and particulate ^{231}Pa and ^{230}Th in the Atlantic Ocean: constraints on intermediate/deep water age, boundary scavenging, and $^{231}Pa/^{230}Th$ fractionation. *Earth Planet. Sci. Lett.* **203**, 999–1014 (2002).
20. Hoffmann, S. S., McManus, J. F., Curry, W. B. & Brown-Leger, L. S. Persistent export of ^{231}Pa from the deep central Arctic Ocean over the past 35,000 years. *Nature* **497**, 603–606 (2013).
21. Vivancos, S. M. *et al.* Inter-Basin Comparison of Boundary Scavenging Expressed in Dissolved and Particulate Thorium and Protactinium Along GEOTRACES GN01, GP16, and GA03 Transects. in *Ocean Sciences Meeting* (2020).
22. Carmack, E. C., Macdonald, R. W. & Jasper, S. Phytoplankton productivity on the Canadian Shelf of the Beaufort Sea. *Mar. Ecol. Prog. Ser.* **277**, 37–50 (2004).
23. O'Brien, M. C., Macdonald, R. W., Melling, H. & Iseki, K. Particle fluxes and geochemistry on the Canadian Beaufort Shelf: Implications for sediment transport and deposition. *Cont. Shelf Res.* **26**, 41–81 (2006).
24. Rudels, B. *et al.* Circulation and transformation of Atlantic water in the Eurasian Basin and the contribution of the Fram Strait inflow branch to the Arctic Ocean heat budget. *Prog. Oceanogr.* **132**, 128–152 (2015).
25. Djinoridze, R. N., Ivanov, G. I., Djinoridze, E. N. & Spielhagen, R. F. Diatoms from Surface Sediments of the Saint Anna Trough. in *Land-Ocean Systems in the Siberian Arctic: Dynamics and History* (eds. Kassens, H. et al.) 553–560 (Springer-Verlag, 1998).
26. Shevchenko, V. P., Ivanov, G. I. & Zernova, V. Vertical particle fluxes in the St. Anna Ttrough and in the Eastern Barents Sea in August-September 1994. in *Modern and Late Quaternary Depositional Environment of the St. Anna Trough Area, Northern Kara Sea* (eds. Stein, R., Fahl, K., Ivanov, G. I., Leviten, M. A. & Tarasov, G.) 46–64 (1999).
27. Luo, Y. & Lippold, J. Controls on ^{231}Pa and ^{230}Th in the Arctic Ocean. *Geophys. Res. Lett.* **42**, 5942–5949 (2015).
28. Gdaniec, S. *et al.* ^{231}Pa and ^{230}Th in the Arctic Ocean: Implications for boundary scavenging and ^{231}Pa-^{230}Th fractionation in the Eurasian Basin. *Chem. Geol.* **532**, 119380 (2020).
29. Fahl, K. & Nöthig, E. M. Lithogenic and biogenic particle fluxes on the Lomonosov Ridge (central Arctic Ocean) and their relevance for sediment accumulation: Vertical vs. lateral transport. *Deep. Res. Part I Oceanogr. Res. Pap.* **54**, 1256–1272 (2007).
