# Supplementary material for: Radioisotope constraints of Arctic deep water export to the North Atlantic
Source: Nat Commun. 2021 Jun 16;12:3658. doi: 10.1038/s41467-021-23877-4 (PMC8209033; doi:10.1038/s41467-021-23877-4)
Supplement: Supplementary file 3 — Description of additional supplementary files [file 41467_2021_23877_MOESM3_ESM.docx]

Description of additional supplementary information

Title: Supplementary Data 1

Description: Locations and radionuclide activities of Arctic Ocean surface sediments.
